# Playfully Assessing Lower Extremity Selective Voluntary Motor Control in Children With Cerebral Palsy: Psychometric Study

**DOI:** 10.2196/39687

**Published:** 2022-12-16

**Authors:** Annina Fahr, Julia Balzer, Jeffrey W Keller, Hubertus J A van Hedel

**Affiliations:** 1 Swiss Children's Rehab University Children's Hospital Zurich Affoltern a.A. Switzerland; 2 Children's Research Center University Children's Hospital Zurich University of Zurich Zurich Switzerland; 3 Institute for Biomechanics ETH Zurich Zurich Switzerland; 4 Doctoral Program Clinical Science Faculty of Medicine University of Zurich Zurich Switzerland

**Keywords:** selective motor control, mirror movements, neurorehabilitation, validity, reliability, interactive computer play, eHealth, digital health, rehabilitation, cerebral palsy, movement, child, pediatric, game, accelerometer, motor, avatar, assessment, limb, joint, physiotherapy, physiotherapist, lower extremity, lower extremities

## Abstract

**Background:**

Objective measures specifically assessing selective voluntary motor control are scarce. Therefore, we have developed an interval-scaled assessment based on accelerometers.

**Objective:**

This study provided a preliminary evaluation of the validity and reliability of this novel gamelike assessment measuring lower limb selective voluntary motor control in children with cerebral palsy (CP).

**Methods:**

Children with CP and their neurologically intact peers were recruited for this psychometric evaluation of the assessgame. The participants played the assessgame and steered an avatar by selective hip, knee, or ankle joint movements captured with accelerometers. The assessgame’s scores provide information about the accuracy of the selective movement of the target joint and the amplitude and frequency of involuntary movements occurring in uninvolved joints. We established discriminative validity by comparing the assessgame scores of the children with CP with those of the neurologically intact children, concurrent validity by correlations with clinical scores and therapists’ opinions, and relative and absolute test-retest reliability.

**Results:**

We included 20 children with CP (mean age 12 years and 5 months, SD 3 years and 4 months; Gross Motor Function Classification System levels I to IV) and 31 neurologically intact children (mean age 11 years and 1 month, SD 3 years and 6 months). The assessgame could distinguish between the children with CP and neurologically intact children. The correlations between the assessgame’s involuntary movement score and the therapist’s rating of the occurrence of involuntary movements during the game were moderate (Spearman ρ=0.56; *P*=.01), whereas the correlations of the assessgame outcomes with the Selective Control Assessment of the Lower Extremity and Gross Motor Function Classification System were low and not significant (|ρ|≤0.39). The intraclass correlation coefficients were >0.85 and indicated good relative test-retest reliability. Minimal detectable changes amounted to 25% (accuracy) and 44% (involuntary movement score) of the mean total scores. The percentage of children able to improve by the minimal detectable change without reaching the maximum score was 100% (17/17) for the accuracy score and 94% (16/17) for the involuntary movement score.

**Conclusions:**

The assessgame proved reliable and showed discriminative validity in this preliminary evaluation. Concurrent validity was moderate with the therapist’s opinion but relatively poor with the Selective Control Assessment of the Lower Extremity. We assume that the assessment’s gamelike character demanded various other motor control aspects that are less considered in current clinical assessments.

## Introduction

### Background

A loss of selective voluntary motor control (SVMC) is a common negative sign in patients with lesions of the upper motor neuron, for instance, children with cerebral palsy (CP) [1,2]. A reduction in SVMC is defined as the impaired ability “to isolate the activation of muscles in a selected pattern in response to demands of a voluntary posture or movement” [1]. Thus, reduced SVMC manifests as involuntary movements that accompany a voluntary movement. Impaired SVMC belongs to the International Classification of Functioning, Disability, and Health Core Sets for Children and Youth with CP alongside other common impairments in this patient group (eg, spasticity, contractures, and muscle weakness) [3]. In comparison to these impairments, reduced SVMC seems to limit other body functions such as muscle strength or activities such as walking [4-8].

Despite the well-known importance of lower limb SVMC for motor activities, only a few tools are used to measure it [9]. According to the systematic review by Balzer et al [10], only 7 assessment tools for the selectivity of single-joint movements of the lower extremities have been tested for their psychometric properties in children with upper motor neuron lesions. The Selective Control Assessment of the Lower Extremity (SCALE) is considered to have the best properties. Its ordinal scoring system with 3 levels (normal, impaired, and unable) is likely to be able to classify SVMC impairments. Nevertheless, the sensitivity of the SCALE in detecting small (therapy-induced) changes could be low because of the relatively broad ordinal scoring system [10]. To address this problem, we aimed to measure SVMC more precisely on an interval-based scale and created a playful computer assessment game (“assessgame”) based on accelerometers that is attractive for (young) patients [11]. It assesses SVMC in terms of both the accuracy of a selective movement of the target joint and the amplitude and frequency of involuntary movements occurring. With involuntary movements, we refer to all unintended movements that co-occur with the performance of a voluntary task (eg, mirror movements or abnormal movement synergies) [12]. A first evaluation of the assessgame and the algorithms to process the data showed that the assessgame could be a valid approach to quantify SVMC in a more attractive manner [11]. Moreover, psychometric testing showed that the assessgame is valid and reliable to measure upper extremity SVMC [13]. The assessgame metrics correlated with the Selective Control of the Upper Extremity Scale (an assessment similar to the SCALE but for the upper limbs), with higher correlation coefficients for average scores over all joints (accuracy ρ=−0.37, involuntary movement score ρ=−0.55; all *P*<.05) than for individual joints (0.04<|τ|<0.52). The assessgame discriminated well between patients with upper motor neuron lesions and healthy children. Its relative reliability was good with intraclass correlation coefficients (ICCs) >0.75 for all average scores.

### Objective and Hypotheses

The focus of this study was to perform a similar preliminary investigation of several psychometric properties of the assessgame for the lower limbs in children with CP. As a gold standard is lacking for measuring SVMC, we evaluated the discriminative and concurrent validity. In line with the findings for the upper limbs [13], we hypothesized that the assessgame scores would differ significantly between children with CP and neurologically intact age-matched participants. We expected the differences between patients and their healthy peers to increase with age because healthy young children may still show signs of reduced SVMC (eg, mirror movements) that recede with age [12].

For concurrent validity, we expected moderate to high correlations (0.50≤|ρ|≤0.70) between the assessgame outcomes and the SCALE. In addition, we expected low correlations (|ρ|<0.50) with the Gross Motor Function Classification System (GMFCS), as it is not a specific measure of SVMC. We expected high positive correlations (|ρ|>0.70) of the assessgame scores with a therapist’s rating of movement selectivity during the game to internally validate the analysis algorithm.

Finally, we investigated the test-retest reliability of the assessgame. We considered test-retest reliability as good when ICCs exceeded 0.75 and absolute measurement errors were acceptable.

## Methods

### Participants

Inpatients and outpatients of the Swiss Children’s Rehab of the University Children’s Hospital Zurich were recruited by convenience sampling from June 2017 to March 2018. Inclusion criteria comprised a clinical diagnosis of predominantly spastic CP (ie, unilateral or bilateral spastic CP or mixed CP with distinct spastic components), an age between 6 and 18 years, and the ability to follow simple verbal instructions. Children with a primarily dystonic or ataxic impairment, those with an unstable situation regarding their tonus-regulating medications, or those who had a botulinum toxin injection within the last 6 months or any surgical correction of the lower extremity within the last year were excluded.

For establishing discriminative validity, neurologically intact children aged between 6 and 18 years were recruited. Only children without any medical history of neurological or orthopedic diagnosis within the lower extremity were included. In addition, we recruited neurologically intact adults because the algorithm for the accelerometer data analysis [11] relates children to adult references who have fully complemented the maturation of SVMC to create the final score. The inclusion criteria for this reference group were age between 18 and 50 years, no symptoms in terms of any central or peripheral neurological injury, and no surgery of the lower limbs within the last year. An upper age limit was selected because involuntary movements were shown to increase with age [12,14].

### Ethics Approval

The study was conducted in accordance with the necessary guidelines and approved by the ethical committee of the Canton of Zurich (Nr PB_2016_01843). A member of the study team explained the study to the participants and their parents and provided them with written participant information in age-adapted versions. Sufficient time was provided to reach a decision. Formal consent was obtained before any measurements were conducted. All the participants provided oral informed consent, and written informed consent was obtained from the adults, adolescents aged ≥14 years, and minors’ parents. They were further informed that they may withdraw from the study at any time and that the withdrawal of consent will not affect the participant’s subsequent medical treatment at the Swiss Children’s Rehab.

### SVMC Assessgame

**Figure 1:**
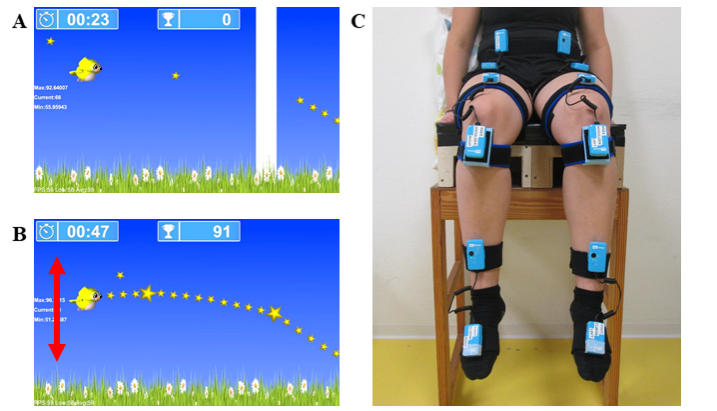
Measurement setup of the assessgame “Catch the Stars.” (A and B) Screenshots from the assessgame showing the owl avatar following and collecting the stars on the target trajectory. (C) Standardized testing position and placement of 6 accelerometer pairs (master-slave) that were fixed proximally and distally to each tested joint.

On the basis of a previous publication [15], we refined the single-joint SVMC-testing concept that was at the base of the development of our assessgame “Catch the Stars.” The assessgame measures SVMC by capturing (accurately) controlled target joint movements and (potentially) simultaneously occurring involuntary movements. The target movements for which SVMC can be measured with the assessgame encompass hip, knee, and ankle flexion and extension. Detailed methodological information about the assessgame can be found in our methodological paper [11]. In short, the participants had to steer an owl avatar on a predefined path made up of stars by the isolated movement of 1 selected target joint. The path consisted of upward and downward curves, lasted 30 seconds, and was presented on a screen placed in front of the participants (Figures 1A and 1B). Six pairs of 3D accelerometers were positioned bilaterally over the hip, knee, and ankle joints (Reha-Stim Medtec AG; Figure 1C). The accelerometer sensors were applied proximally (reference sensor) and distally of the joints to ensure that only movements of those particular joints but not compensatory movements influenced the avatar’s motion. The game was calibrated to the participant’s maximum active range of motion (ROM) of the selected target joint. For the assessment, the participants had to move within 90% of their maximum active ROM.

The participants performed 3 try-out trials (hip, knee, and ankle joints once on a self-selected side) to get familiarized with the game. During the actual measurement, each possible joint was selected once as the target joint (ie, resulting in a total of 6 trials) in a randomized order to control for possible learning effects. After completing the calibration, each game round started with an accommodation phase lasting 25 seconds. During this time, the children could familiarize themselves with steering the avatar by collecting a few stars at different positions on the screen. Immediately at the end of the accommodation phase, which was visualized by a starting line, the star-studded path, that is, the target trajectory, began (Figures 1A and 1B). The participant was instructed to follow this trajectory as accurately as possible with the avatar to collect the stars by only moving the target joint and no other joints. The test phase ended with crossing the finish line after 30 seconds.

To quantify how selectively the game was played, we calculated offline for each target joint an accuracy score and an involuntary movement score that included all simultaneously occurring movements in contralateral or adjacent joints or the trunk (refer to the study by Keller et al [11] for details). First, the data recorded during the assessgame were imported to Matlab (Matlab 2016a, The MathWorks Inc). The accelerometer data were transformed to joint angles, and the time derivative was calculated to yield the angular joint speeds (angle/second). We were interested in these changes in joint angles and not in the absolute position because they represent the movements that occurred while playing the game. We replaced occasionally missing data points (0.8%) because of undetected breakdowns of 1 sensor with the mean of 50 simulated values using multiple imputation by chained equations. For detailed information, refer to the supplementary material of our methods paper [11].

Then, we calculated the accuracy score and the involuntary movement score, which we standardized to the reference values of 31 neurologically intact adults representing movement mastery. The accuracy score represents the standardized error value (standardized to the SD of neurologically intact adults) between the actual trajectory of the avatar and the target path. It displayed how well the participant could move the target joint to follow the target path accurately. The involuntary movement score describes for all nontarget joints the average difference of the joint movement (angular joint speed) of the participant from the adult mean and is expressed in adult SD units. Larger values suggest worse selective control for both the outcomes.

### Comparator Measures

The first comparator assessment was the SCALE, a valid and established clinical assessment of lower limb SVMC [16]. It requires the child to perform specific and timed individual reciprocal joint movements (in this study, the hip, knee, and ankle joints). According to the grading criteria, a therapist classifies the impairments of SVMC during these movements on an ordinal 3-point scale. Each joint can be scored as 0=unable, 1=impaired, or 2=normal SVMC. We used the validated German version of the SCALE [17], and it was always rated by the same physiotherapist based on a video recording.

As a second comparator measure, we used the GMFCS, which classifies the functional abilities and limitations in the gross motor function of children with CP, emphasizing on sitting, transfers, and mobility [18]. It focuses on the children’s performance in their habitual environment rather than their capacity in a standardized setting. Functional limitations and the need of assistive technology for mobility are described with 5 levels. Level 1 describes children who walk without restrictions, whereas the self-mobility of children with GMFCS level 5 is severely limited.

The third comparator measure was the physiotherapist’s expert opinion. She rated the occurrence of involuntary movements during the assessgame by evaluating the video recordings afterward. Possible types of involuntary movements were mirror movements, trunk movements, or movements in any other joint. For the analysis, we assigned 1 point for each type of involuntary movement that occurred at least once; for example, if all 3 types of involuntary movements were observed, 3 points were given. A selectively performed movement was assigned 0 points. This therapist rating of involuntary movements occurring during the assessgame (ie, not during the SCALE) allows a simple validation of the algorithm extracting involuntary movements from the accelerometer data.

### Measurements

The standardized measurement procedure was carried out by 2 people out of a team of 3 testers (1 experienced neuropediatric physiotherapist and 2 human movement scientists) within 1 hour per session. The entire measurement was performed in a sitting position on a custom-made wooden seat to standardize the body position. For testing ankle movements, the active lower leg was placed on a support with the knee extended at 30°. First, the SCALE assessment was performed. Then, the participants were equipped with the accelerometers, the try-out trials were performed, and the actual measurements took place.

To evaluate the test-retest reliability of the assessgame, the measurement was repeated by the same team under similar conditions (time of day and room). Inpatients who received intensive multimodal rehabilitation were measured again within 1 week to ensure that they remained stable. Outpatients who received no intensive therapy were measured again within 3 weeks.

### Statistical Analysis

For all outcomes, we calculated the means of the joints on the more and less affected side as well as an overall mean. If a joint could not be tested with the assessgame (eg, too small ROM), we also excluded the corresponding SCALE score from the analysis. Shapiro-Wilk tests and visual inspection of the data showed that most scores were not normally distributed. Therefore, we applied robust methods to test our a priori formulated hypotheses.

Discriminative validity was determined by a robust, bootstrapped analysis of covariance [19] to compare the assessgame scores between the children with CP and their neurologically intact peers at predefined ages of 9.5, 12.5, and 15.5 years (number of bootstrap samples=2000, span parameter=0.7, data were not trimmed, and CIs were adjusted for multiple comparisons).

Concurrent validity was evaluated by correlating the 2 assessgame outcomes (accuracy and involuntary movement scores) with (1) the SCALE score and (2) the GMFCS level. Furthermore, the game’s involuntary movement score was correlated with the therapist’s rating. For summary scores (total and leg means), we calculated Spearman rank correlation coefficients, whereas we used Kendall Tau-b rank correlation coefficients for individual joints, where we expected a high number of ties in the data. The magnitudes of the correlation coefficients were interpreted as negligible (0.00≤|r|≤0.29), low (0.30≤|r|≤0.49), moderate (0.50≤|r|≤0.69), high (0.70≤|r|≤0.89), or very high (|r|≥0.90) [20].

Relative test-retest reliability was investigated using a 2-way random effects model based on absolute agreement (ICC 2,1 according to Shrout and Fleiss nomenclature [21]). To account for nonnormally distributed data, we calculated bias-corrected and accelerated bootstrap 95% CIs (number of bootstrap samples=1000) [22]. ICCs and their corresponding CIs were interpreted according to the guidelines of Koo et al [23]: ICCs <0.50 indicate poor reliability, those between 0.50 and 0.75 indicate moderate reliability, those between 0.75 and 0.90 indicate good reliability, and those >0.90 indicate excellent reliability. Absolute reliability was determined by the SE of measurement (equation 1, σ_t_=variance of trial and σ_e_=variance of residual [random] error) and the minimal detectable change (MDC) at a 95% confidence level (MDC_95%_; equation 2) [24].



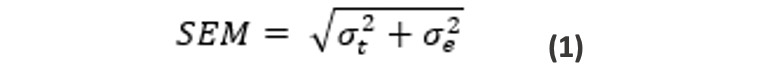





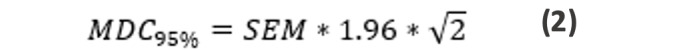



All statistical analyses were performed with R statistical package (version 3.4.4; R Foundation for Statistical Computing) [25] using the additional packages boot version 1.3-20 [26], ICC version 2.3.0 [27], mice version 3.3.0 [28], and WRS2 version 0.10-0 [19]. The significance level was set at α=.05 (2-tailed).

## Results

### Participants’ Characteristics

A total of 24 children with CP provided informed consent. As 4 children were not able to complete the assessgame because of a lack of cognitive understanding of the game (3/4, 75%) or visual impairment (1/4, 25%), we included the data of 20 children with spastic and mixed types of CP (bilateral: 17/20, 85%; unilateral: 3/20, 15%).

Their age ranged from 7 years and 11 months to 17 years and 5 months with a mean age of 12 years and 5 months (SD 3 years and 4 months). Of the 20 participants, 7 (35%) were female. In total, 35% (7/20) of children had GMFCS level I, 15% (3/20) had GMFCS level II, 25% (5/20) had GMFCS level III, and 25% (5/20) had GMFCS level IV. Descriptive statistics of all SVMC measures are presented in Table 1.

A descriptive summary of the peer control group (neurologically intact children: n=31; mean age 11 years and 1 month, SD 3 years and 6 months; n=16, 52% females) and adult reference group (n=31; mean age 33 years and 9 months, SD 7 years and 5 months; n=15, 48% females) is shown in Multimedia Appendix 1.

**Table 1:** Descriptive statistics of the outcome measures.

		More affected leg^a^		Less affected leg		Total	
		Median (IQR^b^)	Range	Median (IQR^b^)	Range	Median (IQR^b^)	Range
**Assessgame**							
	Accuracy	2.42 (1.91-3.20)	0.82-5.52	2.20 (1.63-3.88)	0.81-5.84	2.55 (1.65-3.38)	0.81-5.03
	Involuntary movements	1.65 (1.34-2.65)	0.82-3.58	1.74 (1.23-2.44)	0.75-4.13	1.73 (1.27-2.79)	0.78-3.16
SCALE^c^		1.0 (1.0-1.66)	0.0-2.0	1.33 (1.0-1.75)	0.0-2.0	1.2 (1.0-1.66)	0.0-2.0
Therapist’s opinion		0.83 (0.62-2.00)	0.00-3.00	1.17 (0.46-1.88)	0.00-3.00	1.00 (0.50-1.88)	0.00-3.00

^a^The summary scores (for each leg and total) represent the average values of the individual joints, n=20.

^b^1st-3rd quartile.

^c^SCALE: Selective Control Assessment of the Lower Extremity.

### Discriminative Validity

**Figure 2:**
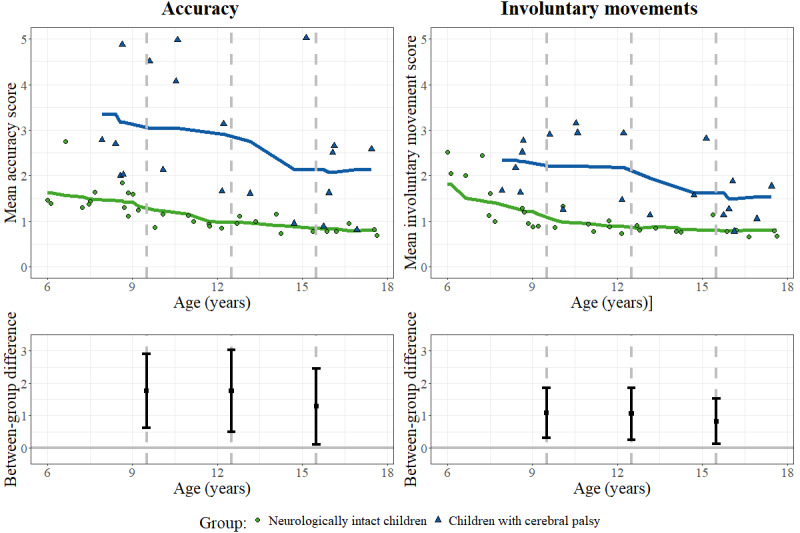
The assessgame scores and age. Scatterplots of the assessgame outcomes by age with smoothing lines are shown for children with cerebral palsy and neurologically intact children separately. Dashed lines depict at which age the patients and neurologically intact children were compared with a robust analysis of covariance. The differences between the groups and the bootstrapped 95% CIs are presented below.

Concerning our hypothesis on discriminative validity, a robust analysis of covariance compared the assessgame total scores between the children with CP and neurologically intact children at the discrete ages of 9.5, 12.5, and 15.5 years. The bootstrapped CIs for the mean difference did not include 0 in any of the comparisons, indicating that the children with CP had significantly worse SVMC (ie, higher scores) than their neurologically intact peers (Figure 2). The group differences were similar across all ages.

### Concurrent Validity

The correlations between the assessgame outcomes and the SCALE and between the assessgame outcomes and GMFCS were negligible to low and nonsignificant (Table 2). Significant correlations of moderate magnitude were found between the involuntary movement score and the therapist’s opinion (Table 2). Correlations with the therapist’s rating were also significant on individual joint level for the ankle and knee (0.47≤τ≤0.74). Complete results for all the individual joints are presented in Multimedia Appendix 1.

**Table 2:** Relationships between the assessgame outcomes and the comparator measures.

		More affected leg			Less affected leg			Total	
		ρ	*P* value	ρ		*P* value	ρ		*P* value
**SCALE^a^**									
	Game accuracy	−0.25	.28	−0.14		.57	−0.29		.21
	Game involuntary movements	−0.19	.43	−0.19		.42	−0.14		.54
**GMFCS^b^ level**									
	Game accuracy	0.42	.06	0.30		.19	0.39		.09
	Game involuntary movements	0.18	.45	0.24		.32	0.23		.32
**Therapist’s opinion**									
	Game involuntary movements	0.59	.006	0.57		.009	0.56		.01

^a^SCALE: Selective Control Assessment of the Lower Extremity.

^b^GMFCS: Gross Motor Function Classification System.

### Test-Retest Reliability

Of the 20 participants, 3 (15%) children could not participate in a second measurement owing to organizational issues. Inpatients were reassessed 1 to 8 days (mean 5.3, SD 2.5 days) after the first appointment, and outpatients were reassessed 6 to 21 days (mean 14.9, SD 6.5 days) later.

With ICC values for the total scores ≥0.86 and the 95% CIs >0.75, the test-retest reliability was in a good range (Table 3). The ICCs for each leg fell into the range of moderate to good test-retest reliability, whereas the CIs of the ICCs for involuntary movement scores were wide. The MDCs listed in Table 3 appeared to be smaller for the accuracy score than for the involuntary movement score and corresponded to 25% to 99% of the mean patient score. The percentage of children (out of 17) that could improve (ie, reduce their assessgame score) by the MDC without surpassing 0 (ie, theoretically, the best possible score) was 100% (n=17) and 94% (n=16) for the total scores (accuracy and involuntary movements, respectively), 82% (n=14) and 29% (n=5) for the more affected side, and 71% (n=12) and 82% (n=14) for the less affected side.

**Table 3:** Test-retest reliability of the assessgame of selective voluntary motor control.

	More affected leg		Less affected leg			Total	
	Game AS^a^	Game IMS^b^	Game AS	Game IMS	Game AS		Game IMS
Mean (SD) 1	2.60 (1.34)	1.88 (0.84)	2.49 (1.48)	1.86 (0.84)	2.55 (1.35)		1.87 (0.73)
Mean (SD) 2	2.62 (1.47)	2.09 (1.19)	2.33 (1.35)	1.62 (0.68)	2.47 (1.35)		1.84 (0.84)
*P* value^c^	.78	.06	.99	.89	.75		.55
ICC^d^ (2,1) (95% CI)	0.94 (0.80-0.98)	0.52 (0.06-0.85)	0.87 (0.57-0.96)	0.80 (0.17-0.93)	0.97 (0.88-0.99)		0.86 (0.76-0.93)
MDC_95_^e^	0.97	1.97	1.40	0.98	0.63		0.82
MDC_95_/grand mean^f^ (%)	37	99	58	56	25		44

^a^AS: accuracy score.

^b^IMS: involuntary movement score.

^c^Uncorrected *P* value of Wilcoxon signed-rank test for systematic differences between the test and retest assessgame scores.

^d^ICC: intraclass correlation coefficient; the *P* values of intraclass correlation coefficients were all <.001, except for the involuntary movement score of the more affected leg (*P*=.008).

^e^MDC_95_: minimal detectable change at 95% confidence level.

^f^The grand mean was the average of the first and second means.

## Discussion

### Principal Findings

We investigated the discriminative and concurrent validity and test-retest reliability of a gamelike assessment for SVMC in children with CP. In summary, the assessgame could differentiate well between the neurologically intact children and children with CP. Although the assessgame’s involuntary movement sum scores correlated moderately with the therapist’s expert opinion about the involuntary movements that occurred during the game, these correlations were high for the ankle and knee joints of the more affected side. The assessgame’s accuracy and involuntary movement scores correlated worse with the SCALE score and the GMFCS level as hypothesized. Test-retest reliability was generally good to excellent, and most ICCs exceeded the minimum required threshold of 0.75, except for the occurrence of involuntary movements of the more affected leg. The acceptability of the absolute reliability was more challenging to interpret, but a high percentage of patients would be able to improve their total scores by the MDC without showing a ceiling effect. When interpreting the scores for the more and less affected sides separately, it seemed that most children with CP could improve in accuracy. At the same time, only some children might be able to reduce the occurrence of involuntary movements. The poorer reliability of the involuntary movement score of the more affected leg might indicate that such movements occur less regularly.

### Discriminative Validity

The assessgame could differentiate well between neurologically intact children and children with CP, with similar differences independent of age. Although we had expected smaller differences in the assessgame scores between young (ie, aged 6-7 years) patients and their neurologically intact peers, our data could not confirm this, as we were not able to recruit children with CP in this age range (Figure 2). When interpreting the running interval smoother in Figure 2 qualitatively, a maturation effect in neurologically intact children can be observed, particularly for the involuntary movement score.

### Concurrent Validity

A comparison with other psychometric studies is only partly possible, as only a few other SVMC tools exist, and their psychometric properties have rarely been investigated [10,16]. When the same assessgame was applied to the upper limbs, the correlations also were the strongest between the assessgame outcomes and the therapist’s rating and weaker between the assessgame outcomes and clinical SVMC measures or classifications of the severity of the disability [13]. However, their absolute correlation coefficients between the assessgame scores and the upper limb equivalent of the SCALE, the selective control of the upper extremity scale, were higher (ie, ρ=−0.37 for accuracy and −0.55 for involuntary movements). Several factors might explain this difference. First, the assessgame for the upper extremity considers a higher number of *df*, allowing more possibilities for involuntary movements than the assessgame for the lower extremities. Second, playing a computer game with the upper extremities is more common, whereas steering an avatar with isolated hip, knee, or foot movements was a new experience for the participants. Third, the assessgame asked for fine-tuned movements. Despite some leg muscles such as the tibialis anterior receiving direct corticospinal projections [29,30], which would allow fine motor control, the lower limbs are generally involved in gross motor movements concerning weight bearing, posture, and ambulation.

The relationships with comparator measures were also weaker than those observed in other lower extremity studies. Although other studies using clinical lower extremity SVMC assessments such as the selective motor control scale or the SCALE found relationships with gross motor function [6,8,16,17,31], the assessgame outcomes did not correlate with the GMFCS level or the SCALE. We expect that this is caused by the differences between the assessgame and clinical assessments of SVMC. First, strength might influence these SVMC measures differently. As the SCALE scores depend on whether the child can actively move through the entire passive ROM, the SCALE correlated strongly with lower limb strength [17,32], which is again a strong prerequisite for walking. By calibrating the assessgame to the active ROM, we aimed to minimize the effect of strength. Second, although the assessgame and a measure such as the SCALE rely on a common definition of SVMC, the assessgame represents a more advanced task requiring graded joint movements of varying amplitudes and speeds. On the basis of the nature of the assessgame, we think that playing the game involved additional visuomotor coordination, action planning, anticipation, and higher cognitive functions. These are all body functions known to vary highly in children with CP [33].

Furthermore, during the measurements, we observed that the children were quite immersed in the assessgame. They focused on collecting as many stars as possible rather than on movement quality (ie, no involuntary movements). Therefore, the assessgame resembles a more playful situation where selective control is required but not the focus of the action. By contrast, during the SCALE, the children had immediate visual control and feedback over their movement, were continuously guided by the therapist, and could display their undivided attention solely on performing selective movements.

Although the relationships between the assessgame outcomes and SCALE were indeed weaker than we had anticipated, the moderate correlations between the assessgame outcomes and therapist’s rating of involuntary movements occurring during the game, which were moderate to high for the knee and high for the ankle joint specifically, indicate that the assessgame is valid in assessing SVMC. The relationships might have been even stronger if the therapist had also quantified the intensity and frequency of the occurring movements.

In our opinion, the assessgame seems to measure SVMC during a more difficult task in a different context (ie, an immersive gaming environment) and in greater detail compared with the current clinical SVMC assessments. It assesses movement control more accurately (ie, finer graded and accurately timed movements) and includes the magnitude and frequency of involuntary movements. These differences make the concurrent validity testing difficult.

### Test-Retest Reliability

As for the reliability results, the English and German versions of the SCALE and the selective motor control scale were shown to have nearly excellent relative reliability in children with CP [16,17,34]. Although we found similarly large ICC values for the total scores in this study, ICCs for the more and less affected sides were lower, especially for the involuntary movement score. We consider 2 explanations. First, although the ordinal clinical scales with only a few levels might mask some possible variability, leading to higher ICCs, the interval-scaled assessgame fully captures this variability. Second, we investigated the test-retest reliability of the assessgame on different test occasions. Previous studies examined the interrater or intrarater reliability by evaluating consecutive assessments on the same day or by rating videotaped assessments. Such protocols might result in smaller variability compared with 2 different test occasions.

The MDCs relative to the grand mean were mostly higher than those found for the SCALE. Balzer et al [17] found values in the range of approximately 30% to 40%. We found comparable values for the total and more affected side accuracy scores, but the values exceeded 40% for the other outcomes. This discrepancy between good relative reliability and rather low absolute reliability can be attributed (in part) to the heterogeneous sample. ICCs can be high even if the trial-to-trial variability is large when between-subject variability is high [35]. The participants, reflecting the population undergoing rehabilitation in our center, varied highly in the level of SVMC impairment as SCALE total scores varied between 1 and 12 out of 12 points. Although these values reflect the reliability one could expect when applying the measure on a daily basis in a heterogeneous clinical population, we expect that for research purposes, more homogeneous patient populations would need to be selected to improve the absolute reliability and allow the assessment of longitudinal changes.

### Limitations

First, the sample size was relatively small; however, with 20 participants, we were sufficiently powered to detect correlations of ≥0.58 (pwr.r.test [36], α=.05, power=80%), which lie clearly below what we expected for the therapist’s opinion and in the lower range of the expected correlations with the SCALE.

Second, the game requires good cognitive functions of participants to maintain concentration and an active ROM of at least 10°; this resulted in dropouts and missing data for some joints. Owing to dynamic or fixed ankle contractures in children with CP, their active ROM was often too small to play the game, and important information on ankle control was lost.

Third, we decided to study children with unilateral and bilateral spastic CP for this preliminary investigation of psychometric properties and usability. Although this sample was more homogeneous than the sample included in the psychometric study of the upper limb assessgame [13], the heterogeneity between patients was large, and the sample represented a population seen in clinics to whom the tool will be applied.

Fourth, reliability could likely be improved by testing each joint more than once and taking the average outcome. Several repetitions would also allow better control for learning effects, if they exist. However, repeating joint assessments seems unfeasible in terms of motivation and compliance if all joints are to be tested. The assessgame was developed to keep motivation and emotional engagement high during testing. Nevertheless, engagement differed between participants and might have influenced the outcomes independent of the participant’s selective control abilities.

Finally, we underestimated the differences in patient requirements for performing the clinical SCALE and the assessgame. Although both assessments build on the definition of reduced selective motor control, the assessgame differs in various aspects, as discussed earlier, which has negatively influenced parts of the validity analyses. Future psychometric studies (on similar assessments) should consider this and could further evaluate the validity of the assessgame by including comparator assessments of more refined control of movement or muscle activation, action planning or visuomotor coordination, and the frequency and amplitude of involuntary movements.

### Clinical Implications

Unlike the SCALE, whose single outcome includes both timed gross motor control and the presence of involuntary movements, the assessgame separates movement accuracy from the occurrence of involuntary movements. This separation is of clinical importance, as it could direct therapists in personalizing the therapy program (eg, improving accurate motor control or inhibiting involuntary movements). However, in particular, the assessgame’s involuntary movement score should become more refined to inform therapeutic decisions in more detail (ie, differentiating between mirror movements and comovements). As a first attempt, we developed a possible clinical output for the assessgame providing information similarly to the descriptors of the SCALE (an example is shown in Multimedia Appendix 2). This output displays in detail how selectively the child was able to play the assessgame for each joint compared with the recorded reference values (neurologically intact adults and control children of approximately the same age as the patient).

The setup, test conduction, and analysis of the assessgame are rather time consuming, which limits the practicability of such an assessment in its current form. Technical adaptations could help optimize the setup such that fewer sensors are required. Furthermore, instead of testing all joints, the test could be focused on 1 or 2 specific target joints selected based on their relevance for the children and their families.

Future studies should investigate SVMC in a larger sample of healthy children to establish robust norm values for the assessgame scores. A first analysis of the current data from 31 healthy children already showed a strong correlation with age [37]. In addition, in a larger sample of children with CP, it might be possible to find SVMC subcategories, which might serve to predict or optimize (physiotherapeutic) treatment output, as has been shown for the SCALE and orthopedic knee surgery [38,39].

Another clinical and scientific application could be to adapt the concept of the assessgame for an intervention to train SVMC in children with CP. Meanwhile, we have developed an interactive computer game for improving SVMC [40]. We took advantage of the assessgame’s motivational effect due to the gaming character and the enriched environment, as these components are indicated to enhance motor learning and neuroplasticity [41,42]. Regardless of which therapeutic approach is taken to improve SVMC, a responsive outcome measure is needed to measure the real changes stemming from interventions. Therefore, future studies might investigate the responsiveness and clinically important changes of the assessgame.

Finally, although we evaluated this assessgame in children with CP, it could be applied to various patient groups with upper motor neuron lesions, both young and adult, as shown for the upper limbs [13].

### Conclusions

This study provided preliminary evidence for the validity and good relative test-retest reliability of a new playful assessgame to measure SVMC of the lower extremities in children with CP. The assessgame differs from the existing assessments of SVMC, and its gaming character might have a complementary value in the measurement of SVMC. This may deepen our understanding of the complex mechanism of motor control. Future studies must show whether and which other aspects of motor control the assessgame includes for it to become an appropriate assessment for clinical or research use. As the relative reliability was good but the absolute reliability was rather low, further studies are needed to investigate the responsiveness of the assessment.

## Appendix

Multimedia Appendix 1 Descriptive statistics of the healthy reference groups, the relationships between the assessgame outcomes and comparator measures for individual joints, and test-retest reliability analyses for individual joints.

Multimedia Appendix 2 Example of a clinical output for the assessgame.

## Abbreviations

CP: cerebral palsy
GMFCS: Gross Motor Function Classification System
ICC: intraclass correlation coefficient
MDC: minimal detectable change
ROM: range of motion
SCALE: Selective Control Assessment of the Lower Extremity
SVMC: selective voluntary motor control

## Data Availability

The data sets generated and analyzed during this study are not publicly available owing to ethical concerns about the small number of patients but are available from the corresponding author upon reasonable request.
