# Supplementary material for: Playfully Assessing Lower Extremity Selective Voluntary Motor Control in Children With Cerebral Palsy: Psychometric Study
Source: JMIR Rehabil Assist Technol. 2022 Dec 16;9(4):e39687. doi: 10.2196/39687 (PMC9804089; doi:10.2196/39687)
Supplement: Multimedia Appendix 1 [file rehab_v9i4e39687_app1.docx]

Supplementary Table 1: Descriptive statistics of the healthy reference groups.

|  | **healthy adults**  **n = 31** | | **healthy children**  **n = 31** | |
| --- | --- | --- | --- | --- |
|  | median [IQR] | range | median [IQR] | range |
| **assessgame** accuracy | 0.77 [0.70,0.89] | 0.62 - 1.08 | 1.12 [0.88,1.38] | 0.69 - 2.75 |
| **assessgame**  invol. movements | 0.78 [0.71,0.83] | 0.65 - 1.12 | 0.91 [0.80,1.17] | 0.66 - 2.52 |
| **SCALE** | NA | NA | 1.83 [1.66,2.00] | 1.00 - 2.00 |

28 of 31 (90.3%) of children and 23 of 31 (74%) of adults indicated the right foot to be the dominant. Abbreviations: invol.: Involuntary; IQR: Interquartile range [1st,3rd quartile]; NA: Not assessed; SCALE: Selective Control Assessment of the Lower Extremity; SD: Standard deviation.

Supplementary Table 2: Relationships between the assessgame outcomes and the comparator measures and test-retest reliability for individual joints.

|  |  |  | **SCALE**  τ (*P*-value) | **Therapist opinion**  τ (*P*-value) | **ICC (2,1)**  **[95%-CI]** | **MDC_95_** |
| --- | --- | --- | --- | --- | --- | --- |
| **game accuracy** | | |  |  |  |  |
|  | **more affected** | |  |  |  |  |
|  |  | Hip | -0.23 (.26) | NA | 0.75 [0.33,0.90] | 2.34 |
|  |  | Knee | -0.11 (.56) | NA | 0.81 [0.37,0.98] | 1.69 |
|  |  | Ankle | 0.18 (.48) | NA | 0.86 [0.75,0.97] | 1.35 |
|  | **less affected** | |  |  |  |  |
|  |  | Hip | -0.04 (.86) | NA | 0.74 [0.49,0.97] | 1.83 |
|  |  | Knee | 0.01 (.94) | NA | 0.85 [0.49,0.95] | 1.51 |
|  |  | Ankle | -0.16 (.55) | NA | 0.80 [0.31,0.97] | 1.27 |
| **game invol. movements** | | |  |  |  |  |
|  | **more affected** | |  |  |  |  |
|  |  | Hip | -0.17 (.40) | 0.26 (.18) | 0.57 [0.05,0.79] | 2.10 |
|  |  | Knee | -0.27 (.15) | 0.62 (<.001) | 0.52 [0.08,0.80] | 1.99 |
|  |  | Ankle | 0.14 (.60) | 0.71 (.005) | 0.68 [0.45,0.86] | 2.12 |
|  | **less affected** | |  |  |  |  |
|  |  | Hip | 0.04 (.86) | 0.15 (.44) | 0.78 [0.55,0.92] | 1.18 |
|  |  | Knee | -0.13 (.47) | 0.47 (.008) | 0.56 [0.00,0.84] | 1.50 |
|  |  | Ankle | -0.21 (.42) | 0.74 (.004) | 0.70 [0.45,0.89] | 0.67 |

The number of data sets for each joint for validity was: Hip n = 18, Knee n = 20, Ankle n = 11 and for reliability: Hip n = 15, Knee n = 17, Ankle n = 9/8 (less/more affected side). Abbreviations: CI: Confidence interval; ICC: Intra-class correlation coefficient; invol.: Involuntary; MDC_95_: Minimal detectable change at 95% confidence; NA: Not assessed; SCALE: Selective Control Assessment of the Lower Extremity.
